# Early outreach to survivors of the shootings in Norway on the 22nd of July 2011

**DOI:** 10.3402/ejpt.v5.23523

**Published:** 2014-07-02

**Authors:** Grete Dyb, Tine Jensen, Kristin Alve Glad, Egil Nygaard, Siri Thoresen

**Affiliations:** 1 Norwegian Centre for Violence and Traumatic Stress Studies, Oslo, Norway; 2 Institute of Clinical Medicine, Faculty of Medicine, University of Oslo, Oslo, Norway; 3 Department of Psychology, Faculty of Social Sciences, University of Oslo, Oslo, Norway

**Keywords:** Posttraumatic stress reactions, PTSD, youth, terror, shootings, early intervention

## Abstract

**Background:**

Under-treatment and unmet needs among survivors have been documented years after terror attacks. Improved early and proactive outreach strategies, including targeted interventions for individuals in need, are required. After the terrorist attacks in Norway on 22 July 2011, a national, proactive outreach strategy was developed and implemented to help those who were directly affected.

**Objectives:**

The aims of this study were threefold: (1) to investigate whether the survivors at the island of Utøya had received proactive outreach from the municipalities, (2) to examine the relationships between received health services and the survivors’ level of exposure and post-trauma health problems, and (3) to explore the level of unmet needs among survivors 5 months post-terror.

**Methods:**

Three hundred and twenty five survivors (*M* age=19.4, SD=4.6, 47.1% females, response rate 66%) of the 2011 massacre on Utøya Island, Norway, were interviewed face-to-face 4–5 months post-terror. The survivors were asked if they had received proactive outreach from their municipality, and what type of health services they had received. Survivors’ level of peri-trauma exposure, loss and injury, posttraumatic stress reactions, symptoms of anxiety and depression, somatic health problems, and sick leave, were assessed.

**Results:**

Most participants (87%) reported that they had received early and proactive outreach, and most (84%) had a contact person. In addition a majority of the survivors has received support from their general practitioner (63%), or other municipal help services (66%). Specialized mental health services by psychiatrists or psychologists had been provided to 73.1% of the survivors. Survivors who had been referred to specialized mental health services reported higher levels of exposure to trauma, posttraumatic stress reactions, depression and anxiety, and somatic health problems, compared to non-receivers of such services. Forty-three survivors (14%) reported unmet needs for services.

**Conclusion:**

In accordance with the national strategy, the vast majority of the participants in this study had received an early and proactive outreach and targeted responses from specialized mental health services had been provided to survivors in need of more extensive help. However, an important minority of the participants had not been reached as planned. The knowledge from this study may guide professionals and decision makers in planning for future disasters and improve the levels of care.

On 22 July 2011, Norway experienced two consecutive terrorist attacks against the government, the civilian population, and an island summer camp hosting members from the governing Labour Party's youth organization. In the first attack, a car bomb was detonated outside the executive government quarter in Oslo, the capital of Norway. The second attack occurred less than 2 hours later at a summer camp on the island of Utøya, with 564 participants. The perpetrator shot, killed, and wounded those he came across. When he was apprehended by the police, 68 had been killed, and one died later in the hospital. Many more were injured, and 56 were hospitalized (The Norwegian Directorate of Health, 2012). Questions immediately arose as to how the shootings would affect the survivors and their families, and how health care authorities should respond.

## Mental health in the aftermath of trauma

A significant number of survivors of shootings experience immediate intense reactions of distress (Neria, DiGrande, & Adams, 2011; Scrimin et al., 2006). Studies investigating long-term trajectories of posttraumatic stress disorder (PTSD) indicate great heterogeneity in post-disaster health development. For example, Bonnano and collaborators (Bonanno, Brewin, Kaniasty, & Greca, 2010) describe four typical trajectories: resilient, healing, chronic, and late-onset developmental patterns. A minority of survivors will develop enduring mental health problems, such as PTSD and depression (Bonanno et al., 2010; Johnson, North, & Smith, 2002; Nader, Pynoos, Fairbanks, & Frederick, 1990; North, Smith, & Spitznagel, 1994; Pynoos et al., 1987; Schwarz & Kowalski, 1991; Trappler & Friedman, 1996). The severity of exposure and subsequent life stress are generally the most important predictive factors for mental health problems after traumatic events, in addition to the emotional reactions during the event, the physical injuries, the loss of close ones, being female, poverty, previous mental health problems, and personality (Brewin, Andrews, & Valentine, 2000; Layne et al., 2010; Neria et al., 2011; Norris et al., 2002; Ozer, Best, Lipsey, & Weiss, 2003). In addition, post-event factors (such as social support), as well as secondary adversities (such as witnessing criminal law trials, involvements in legal claims, extended media coverage of the event, and economic hardships), may be of particular importance (Brewin et al., 2000; Norris et al., 2002).

## Evidence-informed principles for outreach

Under-treatment and unmet needs among survivors have been documented years after terror attacks (Brewin et al., 2010). Improved outreach strategies are required, and in recent years, consensus documents have been developed to provide evidence-informed principles and recommendations for planning and providing outreach post-disaster (Bisson et al., 2010; Hobfoll et al., 2007; National Commission on Children and Disasters, 2010). In these consensus documents, early and proactive outreach to all survivors of mass trauma has been recommended (American Psychiatric Association, 2006). Furthermore, the outreach should provide general support and the necessary resources to ease the transition back to normality, whereas more targeted responses should be provided for individuals in need of more extensive help (O'Donnell et al., 2012; Zatzick et al., 2004). Psychosocial responses should be tailored for each specific disaster, taking into account the type of disaster, the impact on the population, and the structure of existing health care systems (Reifels et al., 2013).

## The 22nd of July outreach strategy

Within days after the terror attack in Norway, county governors received feedback suggesting that survivors’ needs were not being met in the health system. As the survivors from the summer camp at Utøya represented all the 19 Norwegian counties, a national plan was called for, and the Norwegian Center for Violence and Traumatic Stress Studies (NKVTS) was asked by the Secretary of Health to develop a national outreach strategy. On 28 July, the recommendations developed by NKVTS were approved as the governmental strategy and implemented in the affected municipalities. The outreach program was based on three main principles: proactivity in early outreach, continuity in responses, and targeted interventions for individuals in need. The main features of the program were as follows: The crisis teams in the municipalities were queried to establish early contact with the survivors and their families. A designated individual in the municipality was appointed the role of “contact person” for the survivors and their families for at least the first year. The contact person was responsible for monitoring the survivors’ needs and for providing further contact with relevant primary care and mental health services. To aid the contact person in identifying individuals with clinical needs, a simple screening instrument was developed (Helsedirektoratet, 2011). It was recommended that basic screening was to be performed at 5–6 weeks, 3 months and 6 months after the attack. A referral to specialists was recommended if the survivors or their family expressed a need for treatment, the clinical evaluations indicated such a need, or the survivors scored above the clinical cut-off on the screening instrument. Contact persons were informed to be alert for sleep problems, a reduction in functioning level, specific life difficulties, coping with important transitions (e.g., returning to school or work after summer vacation), self-blame, social isolation, sadness, excessive use of alcohol or drugs, and a reluctance to engage in daily activities. The intent of these recommendations was to ensure that all survivors who developed a need for services were identified and offered relevant attention.

## The present study

The aim of this study was to examine whether the recommendations in the national, proactive outreach strategy developed to help the survivors from Utøya had been implemented. More specifically, we wanted to find out whether the survivors had received a proactive outreach from their municipality and to examine the relationship between the services received and the survivors’ levels of exposure and post-trauma health problems. Based on the recommendations in the outreach program, we expected to find that the vast majority of the survivors had been contacted by their municipality, and that survivors who had received specialized services would have a higher symptom load than non-receivers. We also wanted to explore the level of unmet needs among the survivors 5 months after the terror attack.

## Methods

### Subjects

In total, 495 survivors were registered by the police after the terrorist attack on the island of Utøya. In this study, four individuals were excluded because of an age below 13 years, and one individual was living abroad and could not be reached for an interview. Thus, postal invitations were sent out to 490 survivors. Three survivors opted out by sending a text message to the research team. The remaining 487 were contacted by phone. Of them, 135 declined to participate, whereof 55 provided a reason (e.g., they did not have the time or they did not want to stir things up or be reminded of the tragedy); 27 could not be reached by phone. The remaining 325 (66.3%) survivors were interviewed. There were no significant differences between participants and non-responders in age [*M*=19.4 years vs. *M*=19.0, respectively, *t* (488)=0.99, *p*=.32], gender [47.1% female vs. 42.4%, respectively, χ^2^ (490, 1)=0.96, *p*=.33], or where they lived [χ^2^ (490, 5)=6.71, *p*=.24].

### Procedures

Five months after the terrorist attack on Utøya, the survivors received a postal invitation to participate in the study. In the letter, individuals were informed about the study and informed that they would shortly be contacted via phone by an interviewer. All survivors were given the opportunity to opt out by calling or sending a text message to the research team. Most interviews (95.4%) were conducted in November and December of 2011. The participants were interviewed separately, face-to-face, most of them in their homes, but some were interviewed in an alternative location at the request of the participant. The interviews were semi-structured and conducted by health care personnel (psychologists, medical doctors, nurses, or other professionals with a master's degree). Prior to conducting the interviews, the interviewers attended a 1-day training program. The interviews were audio taped and lasted approximately an hour and a half. All participants provided written consent, and the study was approved by the Regional Committees for Medical and Health Research Ethics in Norway.

### Measures

#### Proactive outreach from the municipality

To determine the level of proactive outreach received from the municipality after the terror attack on Utøya, the participants were asked whether they had immediately been contacted after the attack by someone from the crisis team or an alternative municipality service, and whether they had a contact person in the municipality.

#### Provided health services

Contacts with a general practitioner, other health care personnel in primary care services, and a psychiatrist or psychologist in specialized health care services in the aftermath of the terror attack were registered (yes=1, no=0). The perceived usefulness of the health care services was rated on a scale of 1 (not at all), 2 (to some extent), or 3 (very much). The participants were also asked whether their need for help after the terror attack had been met (e.g., by a doctor, psychologist, social worker, or other professionals) (yes=1, no=0).

#### Terror exposure

To determine the youths’ level of exposure during the terror attack, a 14-item checklist was developed. The items were based on critical events experienced on the island and included the following variables: saw the perpetrator or heard his voice; hid from or ran from the perpetrator; heard gun shots; heard people screaming; smelled gun-fire or other distinct smells; saw someone be injured or killed; heard someone be injured or killed; saw dead bodies; touched dead bodies or injured people; was afraid of being seriously injured; was afraid that he/she would die; saw the perpetrator point the gun at him/her, or realized that he had shot at him/her; was afraid that he/she would drown; and felt threatened by the police. The items were rated with a yes/no response scale (yes=1, no=0; range 0–14). The exposure items showed an acceptable level of internal consistency (Cronbach's alpha=.57).

#### Loss and injury

The participants were asked if they had a friend, partner, or family member who died in the terror attacks (yes=1, no=0). The participants were also asked whether they were hospitalized due to injuries (yes=1, no=0).

#### Posttraumatic stress symptoms

The participants were interviewed about their posttraumatic stress reactions at 5 months after the attack using the UCLA PTSD Reaction Index (PTSD-RI) (Steinberg, Brymer, Decker, & Pynoos, 2004). Although the UCLA index is often used as a self-report instrument, in this study, professionals interviewed the participants face-to-face to ensure valid answers.

The PTSD-RI is a 20-item scale assessing posttraumatic stress reactions in the past month. Responses are recorded on a 5-point Likert-scale, ranging from 0 (never) to 4 (most of the time). Three of the items have two alternative formulations, with the highest frequency score used to calculate the total score. Hence, 17 scores constitute the total symptom scale score (possible range 0–68) corresponding to PTSD criteria in the *Diagnostic and Statistical Manual of Mental Disorders* (Fourth Edition) (American Psychiatric Association, 2000). In the present study, the Cronbach's alpha of the total scale was .89.

#### Symptoms of depression and anxiety

To determine the participant's level of depression and anxiety within the previous 2 weeks, an 8-item version (SCL-8) of the Hopkins Symptom Checklist-25 was used (Solberg et al., 2011). Each item was rated on a scale from 1 (not at all bothered) to 4 (very much bothered). Short versions of the SCL-25 have previously been used in Norwegian population surveys and have shown high correlations with the 25-item scale and good psychometric properties (Strand, Dalgard, Tambs, & Rognerud, 2003; Tambs & Moum, 1993). In the present study, the Cronbach's alpha of the total scale was .85.

#### Somatic health problems and sick leave

The level of somatic symptoms within the previous 2 weeks was measured by a short version of the Children's Somatic Symptoms Inventory (CSSI-8) (Walker, Beck, Garber, & Lambert, 2009), including pain in the stomach, head, lower back, and arms or legs; faintness or dizziness, a rapid heartbeat, nausea or upset stomach; and weakness in parts of the body. Each item was rated on a scale from 1 (not at all bothered) to 4 (very much bothered), and the mean scores were calculated. The Cronbach's alpha in the present study was acceptable (.77). The participants’ sick leave from jobs or studies during the past 3 months was registered.

### Statistics

A Pearson chi-square test was used for bivariate analyses of two categorical variables. Student's *t*-test and ANOVA were used for comparing mean differences between two or more groups. Because of the skewed distribution of the number of appointments used in mental health services, a Kruskal–Wallis test of independent samples was used when analyzing the differences between groups in the number of appointments. No participants had more than two missing variables within any sum scores (exposure, PTSD, somatic health problems, or depression and anxiety). Imputations were based on the participants own scores on the other questions within the same sum score. All tests were 2-tailed with a significance level of *p*≤.05. Statistical analyses were performed using IBM SPSS statistics, version 20.

## Results

### Participants

The 325 participants in the study were between 13 and 57 years old (mean=19.4, SD=4.6) at the time of the terror attack; 153 (47.1%) participants were female; 92.5% of the participants were younger than 25 years of age; and 97% were less than 30 years of age. The majority of the participants were youth attending the summer camp; other participants were guests, volunteers, and employees. There were no significant age differences between genders. The participants were highly exposed to danger, with a mean of 9.5 (SD=2.2) out of the 14 exposure items (for detailed information on participants’ exposure, see Dyb et al., 2013). The majority of survivors had lost someone close (*n=*240, 74.5%). A minority of the participants were of non-Norwegian origin (*n=*40, 12.3%). The participants were living in 127 different municipalities that encompassed all 19 counties in Norway. Most survivors were students (*n*=245, 81.1%) and lived with their parents (*n=*202, 63.1%). The majority were members of belief societies: Christian (*n=*202, 63.7%), Islamic (*n*=17, 5.4%), or others (*n*=14, 4.4%).

### Proactive outreach and health care services provided to survivors

Table 1 displays the use of health care services, the frequency of the services provided, and the perceived usefulness of the services at 4–5 months after the terror attack. The vast majority of the participants reported that early and proactive outreach had been provided by the crisis team (*n=*278, 86.9%) and that they had a contact person (*n=*263, 83.8%) in their municipality. No significant differences were found between the receivers of this outreach compared to non-receivers with regard to trauma exposure, loss, mental health status, or demographics such as gender, ethnicity, and age. The participants who had been hospitalized had been contacted by the municipality less often than individuals who had not been hospitalized (73.3% vs. 88.3%, respectively, χ^2^ (320, 1)=5.32, *p=*.02). However, there were significant geographical differences, both for having been contacted by the municipality (χ^2^ (316, 4)=15.45, *p=*.004) and for having a contact person (χ^2^ (310, 4)=18.18, *p=*.001). Although only 3.6% of the participants from Middle Norway reported not having a contact person, 25.2% of the participants from East Norway reported not having a contact person.

**Table 1:** The table displays the use of health care services and perceived usefulness of services reported by survivors 4–5 months after the terror attack (*n=*325)

Provided services	n	%
Psychosocial crisis team (*n=*320)^a^	278	86.9
Contact person (*n=*314)^a^	263	83.8
General practitioner (*n=*320)^a^	200	62.5
Frequency^b^		
≤3	143	71.5
4–10	52	26.0
≥11	1	0.5
Missing	4	2.0
Perceived usefulness^b^		
None	26	13.0
Some	88	44.0
Much	81	40.5
Missing	5	2.5
Other municipal help services (*n=*314)^a^	207	65.9
Frequency^b^		
≤3	83	40.1
4–10	81	39.1
≥11	23	11.1
Missing	20	9.7
Perceived usefulness^b^		
None	29	14.0
Some	86	41.5
Much	86	41.5
Missing	6	2.9
Specialized mental health services (*n=*320^a^	234	73.1
Frequency^b^		
≤3	58	24.8
4–10	115	49.1
≥11	44	18.8
Missing	17	7.3
Perceived usefulness^b^		
None	24	10.3
Some	77	32.9
Much	119	50.9
Missing	14	6.0
Number of services received^c^ (*n=325*)^a^		
0	0	
1	9	2.8
2	34	10.5
3	88	27.1
4	124	38.2
5	69	21.2
Missing	1	0.3

a Number of participants answering the question ranged from 314 to 325 and *n* is displayed for each question.

b Frequency of services and perceived usefulness reported by survivors receiving these services.

c Includes proactive outreach from psychosocial crisis team, contact person in municipality, general practitioner, other municipal help services, and specialized mental health services (count 0–5).

In addition to this outreach, a majority of the survivors reported having used services such as their general practitioner (*n=*200, 62.5%), or other municipal help services (*n=*207, 65.9%). Specialized mental health services by psychiatrists or psychologists had been provided to 73.1% of the survivors (*n=*234). Both the municipality services and the specialized mental health services had provided more frequent consultations than the general practitioners. The majority of the survivors reported services to be somewhat useful or highly useful (Table 1). Most survivors evaluated the usefulness of the specialized mental health services positively (somewhat useful: 35.0%, and very useful: 54.1%). However, an important minority (10.9%) evaluated the services as not useful at all. There were no significant differences between the youth who found the mental health services useful and those who did not with regards to age, gender, level of exposure, and levels of posttraumatic stress reactions or anxiety and depression. However, participants who reported the mental health services to be very useful had received more treatment than those reporting the services to be somewhat useful or not useful at all (mean hours of treatment=9.7, 6.6, and 6.1 hours respectively; *p*<.001).

All survivors in the study had received some type of health service; the majority of the survivors had received help from more than two services (Table 1).

Table 2 displays an overview of the use of municipality services by receivers and non-receivers of specialized mental health services. The receivers of specialized mental health services received less proactive outreach from psychosocial crisis teams, contact persons, and other municipality services, whereas they had more contact with their general practitioner compared to the non-receivers.

**Table 2:** Municipality services provided to receivers and non-receivers of specialized mental health services (*n=*320)

	Receivers (*n=*234)		Non-receivers (*n=*86)				
					
Municipality services	n	%	n	%	*χ* ^2^	df	*p*
Psychosocial crisis team (*n=*316)^a^							
Yes=274	194	83.6	80	95.2	*χ* ^2^=7.22	1	0.01
No=42	38	16.4	4	4.8			
Contact person (*n=*310)^a^							
Yes=260	183	81.0	77	91.7	*χ* ^2^=5.2	1	0.01
No=50	43	19.0	7	8.3			
General practitioner (*n=*317)^a^							
Yes=200	154	66.4	46	54.1	*χ* ^2^=4.0	1	0.05
No=117	78	33.6	39	45.9			
Other municipal help services (*n=*312^a^							
Yes=205	140	61.7	65	76.5	*χ* ^2^=6.0	1	0.02
No=107	87	38.3	20	23.5			

a 320 participants provided information of receiving specialized mental health services. Number of participants providing information of municipality services ranged from 310 to 317.

Targeted responses from specialized mental health services were provided to survivors who were in need of more extensive help. Table 3 displays the prevalence of health problems and the level of peri-trauma exposure in individuals who had received specialized mental health services, compared to individuals who had not, during the first 4–5 months following the terror attack. The survivors who had received specialized services had significantly higher levels of trauma exposure, PTSD symptoms, general mental health problems, somatic health complaints, and a somewhat lower level of sick leave (*p=*0.06), compared to the non-receivers.

**Table 3:** Characteristics of survivors receiving specialized mental health service compared to those who did not (*n=*320).

	Receivers (*n=*234)		Non-receivers (*n=*86)				
					
Characteristics	*n* (%)	mean (SD)	*n* (%)	mean (SD)	*t χ* ^2^	df	*p*
Age							
°*M=*19.4 (SD=4.6)	19.5	5.1	19.1	2.9	*t=*0.71	318	0.48
Gender							
°Female (*n=*151)	114	48.7	37	43.0	*χ* ^2^=0.82	1	0.38
°Male (*n=*169)	120	51.3	49	57.0			
Norwegian origin							
°Yes (*n=*281)	202	86.3	79	91.9	*χ* ^2^=1.80	1	0.25
°No (*n=*39)	32	13.7	7	8.1			
Sum of exposure							
°*M=*9.5 (SD=2.2)	9.6	2.3	9.0	1.9	*t=*2.23	318	0.03
Loss (close friend, partner, family)^a^							
°Yes (*n=*238)	180	77.3	58	69.0	*χ* ^2^=2.22	1	0.14
°No (*n=*79)	53	22.7	26	31.0			
Hospitalized							
°Yes (*n=*30)	25	10.7	5	5.8	*χ* ^2^=1.76	1	0.20
°No (290)	209	89.3	81	94.2			
Level of posttraumatic stress reactions							
°*M=*1.6 (SD=0.7)	1.7	0.7	1.3	0.6	*t=*3.63	318	<0.001
Level of depression/anxiety							
°*M=*2.1 (SD=0.7)	2.2	0.7	1.9	0.7	*t=*3.22	318	0.001
Level of somatic health problems							
°*M=*1.7 (SD=0.5)	1.8	0.6	1.6	0.4	*t=*3.39	318	0.001
Sick leave^b^							
°Yes (*n=*227)	173	76.2	54	65.1	*χ* ^2^=3.91	1	0.06
°No (*n=*86)	56	23.8	30	34.9			

a *n=*317 due to missing data.

b *n=*313 due to missing data.

### Unmet needs

At the end of the interview, the survivors were asked to evaluate if they were currently receiving sufficient help and assistance; 13.7% (*n=*43) of individuals reported not receiving sufficient help related to their adjustment following the terror attack. Reporting an unmet need was not significantly associated with gender, age, loss, hospitalization, sick leave, or the use of health services. However, individuals who reported unmet needs were more often zof non-Norwegian origin [30.0% vs. 8.0%, χ^2^ (302, 1)=17.23, *p<*.001] and reported higher levels of exposure (mean=9.1 vs. 8.3, *t*=2.14, *p*=.03), posttraumatic stress (mean=34.4 vs. 25.2, *t*=4.77, *p*<.001), depression/anxiety (mean=2.3 vs. 1.9, *t*=3.82, *p*<.001), and somatic health problems (mean=2.0 vs. 1.7, *t*=3.26, *p*=.001), compared to survivors reporting no unmet needs of services.

## Discussion

The vast majority of the study participants reported that they had been contacted by the municipality in the early phase following the terror attack and that they had an established contact person. These findings indicate that an early and proactive outreach was implemented in the municipalities in the early phase. Having a contact person was not mandatory, and it may be that some individuals declined this form of help. We did not identify any individual factor that characterized the individuals who did not have a contact person. This is in accordance with the aim of the outreach program, i.e., to provide early proactive outreach unselectively to the survivors in this highly exposed group.

Previous efforts to initiate proactive outreach were made in Norway following the 2004 South East Asian tsunami disaster in which 84 Norwegians were killed and 3,000 individuals survived and returned home (Dyb, Jensen, & Nygaard, 2011; Jensen, Dyb, & Nygaard, 2009).

After the tsunami, it was recommended that the general practitioners contacted and evaluated the survivors’ mental health care needs. Approximately half of the tsunami survivors reported contact with their general practitioners as part of the follow-up, whereas less than 10% reported referrals to specialized mental health services (Hjelmdal, 2007). The findings from the present study suggests that the overall success of the outreach was higher than the proactive outreach following the 2004 tsunami disaster (Dyb et al., 2011; Jensen et al., 2009). This difference in outreach success may indicate that the municipal psychosocial crisis teams were a wiser choice for anchoring the intervention, perhaps because general practitioners already have a heavy work load with their usual patients, whereas the crises teams were assigned this specific task and could devote their time, attention, and resources to the survivors. These teams are specifically trained to reach out to families after tragedy, that is, suicides and accidents. Also, the terror attack made a deep impression on the Norwegian population (Nordanger et al., 2013; Thoresen, Aakvaag, Wentzel-Larsen, Dyb, & Hjemdal, 2012), and the general willingness to help following the terror attack was high. This may have contributed to the municipality's efforts and outreach success.

In contrast to the proactive outreach, the referral to specialized mental health services was a selective intervention. Referrals to such services were recommended if the survivors expressed a need for treatment, the clinical evaluations indicated such a need, or the survivors scored above the cut-off on the screening tool. The finding that the majority (73%) of the survivors had seen a psychologist or a psychiatrist at least once may indicate that the needs were very high, or that the selection procedure was not very strict. As reported elsewhere (Dyb et al., 2013), the survivors in this study had, on average, six times the levels of PTSD compared to the general population, indicating that the need for help was extensive. Consistent with the goal of the outreach program, the results show that the survivors who had received specialized mental health services reported a higher symptom load compared to the survivors who had not received these services. This was true for PTSD symptoms, anxiety and depression, and somatic health complaints, such as headaches and stomach aches. Previous research indicates that the combination of a life threat and the loss of someone close may constitute a greater health risk than a life threat alone (Kristensen, Weisaeth, & Heir, 2009; Neria & Litz, 2004). In our study, the level of loss was high (74.5%), and the loss of someone close was not overrepresented in the group that received treatment.

All services provided were reported to be somewhat or very useful by the majority of survivors. However, the perceived usefulness should not be used as indicative of service outcome. As observed within the research on critical incident stress debriefing, the satisfaction with the intervention may be high even though the mental health benefit is non-existent or negative (Gersons & Olff, 2005). In contrast with stress debriefing, however, a positive perception of mental health assistance may be favorable because it can reduce barriers to seeking help for mental health problems later in life. Data on the survivors’ perceptions of usefulness only represent a snapshot in time, approximately 5 months after the terror attack. Whether these perceptions will change with time may be assessed at future time points in this study.

The majority (86.3%) of survivors reported that the help they received following the terror attack was sufficient. However, the 43 survivors with unmet needs reported more distress and other health problems than the majority of survivors and were more often of non-Norwegian origin. Hence, this group seemed to be in a particularly vulnerable situation despite the outreach program provided. Unmeasured factors, such as the quality of services provided, the survivors’ life situations, the lack of progress in treatment, secondary adversities due to the event, and relationship problems, may have affected the level of needs. The interviewers had instructions to follow-up on unmet needs and arrange for assistance. Currently, we have no systematic data on the outcome of these follow-up procedures. However, the intention was that the research interview would also serve as a safety net for the survivors who had various problems that would benefit from treatment. Interviewers have described a variety of follow-up procedures to meet the needs of the survivors. It is our impression that the use of clinical interviewers who were familiar with local resources was a strength for the intervention strategy and for the acceptability of the study by the survivors, the Norwegian Labour Youth organization, the post-terror self-help organization, and the health authorities.

The data from the interview study 5 months after the terror attack provided an overall positive evaluation of the intervention strategy for those who participated. However, we must not forget that we do not have information on the 34% of the survivors who did not participate in the study, and that an important minority of those who did participate was not reached as planned: Some individuals with a heavy burden of symptoms were not offered treatment, some survivors felt that therapy was not useful to them, and 14% reported unmet needs. We know from other studies that perceived social support tends to decrease with time, and as time goes by, it may not be as easy to mobilize municipalities and specialized services. We may also speculate that the early phases of therapy may be associated with hope and expectations for change and relief. Later measurements may be more characterized by disappointment and resignation for individuals who do not experience the expected changes.

### Strengths and limitations

This study adds significantly to the limited knowledge of early outreach programs by systematically describing the national response program implemented in Norway after the 22nd of July terrorist attack; it describes how the strategy worked on certain key areas. The strengths of this study included the relatively high response rate and the very low levels of missing data. Furthermore, the interviewers were trained professionals, the interviews were performed face-to-face, and the interviewers were provided supervision from the research team throughout the data collection. Survivors in need of professional help were given advice and help in contacting health and social services.

Despite these strengths, our findings must be considered in light of several limitations. The results presented here were correlational; thus, the study was not able to provide conclusions of causality. Also, health services may have been provided to participants for several reasons, not only trauma specific health problems. The experiences of this specific population may provide useful information on the implementation of proactive outreach in countries with similar health care systems, but may not be comparable to the situation in countries with other levels of health services.

It is important to underline that the evaluation of implementation of the outreach program in this paper is based on the experiences reported by those who chose to participate in the study. We do not know what kind of help those who did not participate received or missed. Finally, this study did not measure treatment efficacy or the quality of services provided.

### Practical implications

In sum, in accordance with the national strategy, the vast majority of the participants in this study had received an early and proactive outreach, and targeted responses from specialized mental health services had been provided to survivors in need of more extensive help. However, an important minority of the participants had not been reached as planned.

The knowledge from this study may guide professionals and decision makers in planning for future disasters and improve the levels of care.
